# Monobenzone, a Novel and Potent KDM1A Inhibitor, Suppresses Migration of Gastric Cancer Cells

**DOI:** 10.3389/fphar.2021.640949

**Published:** 2021-04-15

**Authors:** Peizhi Ma, Gang Jia, Zhiyu Song

**Affiliations:** ^1^ Department of Pharmacy, Henan Provincial People's Hospital, People's Hospital of Zhengzhou University, Zhengzhou, Henan, China; ^2^ Department of Oncology, Henan Provincial People's Hospital, People's Hospital of Zhengzhou University, Zhengzhou, Henan, China

**Keywords:** monobenzone, KDM1A inhibitor, gastric cancer, migration, Epithelial–mesenchymal transition, H3K4me1/2, H3K9me1/2, ORY-1001, N-Cadherin

## Abstract

Lysine-specific demethylase1 (KDM1A) is generally highly expressed in various cancer tissues, and promotes the initiation and development of cancers via diverse cellular signaling pathways. Therefore, KDM1A is a promising drug target in many cancers, and it is crucial to find effective KDM1A inhibitors, while none of them has entered into market. With the help of compound library, monobenzone, a local depigmentor using as a treating over-pigmentation in clinic, was characterized as an effective KDM1A inhibitor (IC_50_ = 0.4507 μM), which may competitively inhibit KDM1A reversibly. Further cellular study confirmed that monobenzone could inhibit the proliferation of gastric cancer cell lines MGC-803 and BGC-823 with IC_50_ as 7.82 ± 0.55 μM and 6.99 ± 0.51 μM, respectively, and erase the substrate of KDM1A, H3K4me1/2 and H3K9 me2, and inhibit the migration of gastric cancer cell by reversing epithelial–mesenchymal transition (EMT). As the structure of monobenzone is very simple and small, this study provides a novel backbone for the further optimization of KDM1A inhibitor and gives monobenzone potential new application.

## Introduction

Monobenzone is a potent skin-bleaching agent in melanoma patients (van den Boorn et al., 2011). In 2018, it was also reported that monobenzone was applicable for the treatment of cutaneous melanoma metastases, indicating its additional clinical investigation value (Teulings et al., 2018). Nevertheless, the mechanism of action behind the monobenzone is still unclear, and target of monobenzone has not been clarified. As reported, there is increasing evidence that histone lysine methylation plays a significant role in melanoma metastasis and other solid tumors (Mahmoud et al., 2016; Park et al., 2016; Chen et al., 2020), however, whether monobenzone may act as the regulator and inhibit cell metastasis by regulating histone methylation is not clear.

KDM1A was characterized as the first histone demethylase that can erase mono- and dimethylated lysine 4 and 9 on histone 3 at specific sites using flavin adenine dinucleotide (FAD) as a cofactor (Shi et al., 2004; Maiques-Diaz and Somervaille, 2016). It has been shown to be overexpressed in diverse cancers and contributes to cancer development and progress as an oncogene, thus, inhibition of KDM1A pharmacologically can suppress the proliferation and migration of diverse cancer cells (Zheng et al., 2015; Zheng et al., 2016c; Dai et al., 2020; Jia et al., 2020). Until now, there are plenty of articles that concerning the discovery and development of KDM1A inhibitors (Zheng et al., 2016b; Sun et al., 2017; Xi et al., 2017; Duan et al., 2018; Xi et al., 2018; Xu et al., 2018; Li Z.-R. et al., 2019; Liu et al., 2019), nevertheless, only several of them have entered into clinical trials (Zheng et al., 2015; Zheng et al., 2016; Dai et al., 2020). Thereby, discovery of KDM1A inhibitor with new skeleton is in needed.

In this manuscript, monobenzone was characterized as a reversible KDM1A inhibitor (IC_50_ = 0.4507 μM) from an U.S. Food and Drug Administration (FDA) approved drug library from APExBIO using a drug repositioning strategy. Further molecular docking indicated that monobenzone can penetrate into the active cavity of KDM1A by forming two hydrogen bonds with Arg316 and Thr624 residues. Additional cellular study indicated that KDM1A can bind and inhibit KDM1A in cells and induce the accumulation of KDM1A substrate H3K4me1/2 and H3K9me2. Finally, we found that monobenzone can inhibit gastric cancer migration by reversing epithelial–mesenchymal transition (EMT). All these studies indicated that monobenzone may be considered as a novel skeleton as KDM1A inhibitor for further optimization and used to inhibit gastric cancer cell migration.

## Materials and Methods

### Cell Viability Studies

The MGC-803 and BGC-823 cells were obtained from Cell Bank of Chinse Academy of Sciences at Shanghai, China. Cells were maintained in Dulbecco's Modified Eagle Medium (DMEM) supplied with 10% fetal bovine serum (FBS) in the presence of 100 IU/ml penicillin and 100 μg/ml streptomycin in a 37°C humidified incubator with 5% CO_2_.

The non-radioactive, colorimetric assay system using (3-(4,5-dimethyl-2-thiazolyl)-2,5-diphenyl-2-H-tetrazolium bromide) MTT was performed to evaluate the antiproliferative activity of monobenzone on MGC-803 and BGC-823 cells. First, cells were plated into 96 wells plates at a density of 10^3^ cells per well, then cells were incubated in medium containing diverse concentrations of compounds as indicated. After that, 20 μL MTT was dispensed to each well. After 4 h culture, the supernatant was discarded and 200 μL dimethyl sulfoxide (DMSO) was used to dissolve the insoluble formazan. Finally, the result was read out at 490 nm on the multiplate reader (H1, BioTek, United States).

### KDM1A Assay

The KDM1A assay was performed using a KDM1A Inhibitor Screening Assay Kit from Cayman Chemical (Cayman, United States). Briefly, test compounds were dissolved in 100% DMSO and 10 μL of the diluted drug sample (final concentration at 10 μM), 20 μL horseradish peroxidase (HRP), 140 μL assay buffer, 20 μL KDM1A recombinant and 10 μL fluorometric substrate (10-acetyl-3,7-dihydroxyphenoxazine, named as Amplex Red) were added to a black 96-well plate. Then, 20 μL peptide (dimethyl K4 peptide corresponding to the first 21 amino acids of the N-terminal tail of histone H3) was added to each well except the background well so that to initiate the reaction. After 30 min incubation at 37°C, amount of product (resorufin) was analyzed on an multiplate reader (H1, BioTek, United States) with an excitation wavelength of 530 nm and an emission wavelength of 595 nm.

### Western Blot Analysis

After treatment, cells were collected and and resuspended in RIPA buffer [50 mM Tris HCl pH 8.0, 150 mM NaCl, 1% Nonidet P 40, 0.5% sodium deoxycholate, 0.1% sodium dodecyl sulfate, 1:100 protease inhibitor (Sigma-Aldrich, United States), 1 mM NaV, and 1 mM NaF in H_2_O] on ice for 20 min. Then, the lysate was centrifuged at 12,000 rpm for 15 min at 4°C so that to remove debris. Finally, the supernatant was collected and subjected to BCA assay kit (Beyotime, China) for protein quantification.

Sample was mixed with 1 × SDS loading dye (Bio-Rad, United States) and boiled at 95°C for 10 min. Then this mixture was subjected to 10% SDS-PAGE gel. After running, gel was transferred for 2.0 h at 1000 V, and membrane was blocked with 5% milk in PSBT (0.1% Tween-20 in 1 × PBS) for 1 h at room temperature. Then, membrane was incubated with appropriate primary antibody in PBST at 4°C overnight. Following three times washing with PBST, the membrane was incubated with corresponding secondary antibody for 2 h at room temperature. Then, wash the membrane 3 times for 10 min with PBST again, and get the image with X-ray film with the aid of SuperSignal West Sensitivity Substrate (Thermo Scientific, United States).

### Quantitative Reverse Transcription PCR

After cells were treated as indicated, cells were collected and subjected to RNA extraction (CWBio, China). Then, cDNA was synthesized by cDNA synthesis kit (CWBio, China) with the extracted RNA. qPCR was performed using the SYBR mix (CWBio, China) with under the Applied Biosystems Q6 (USA). The 2^−ΔΔCt^ method was applied to calculate the relative amount mRNA as compared to controls.

### Scratch Assay

Scratch assay was used to examine the wound restoring capacity. Cells were seeded into 24 wells plate (10^5^ cells each well) and grown until reached to 80% confluence. After that, scratches were made using sterile 10 μL pipette tip and cells were re-washed with PBS. Then, media was replaced with serum-free DMEM as well as indicated compound and cells were imaged with microscope (Ts100, Nikon, Japan). After incubating with indicated time, pictures were taken in the same position before and after incubation to document the wound-healing process. Scratch experiments were repeated three times at least and representative pictures are included in this study.

### Tranwell Assay

10^5^ cells were suspended in cell culture medium without FBS, and then seeded into the upper chamber. Meanwhile, 700 µL medium supplied with 20% FBS was used as a chemoattractant and added to the lower chamber. Finally, cells on the upper chamber was removed with swab, and cells on the lower side of the membrane were fixed in 95% ethanol and stained with 2 μg/ml DAPI (Beyotime, China), followed with the analysis by IN Cell Analyzer 6500 HS high content analysis (GE, United States).

### Molecular Modeling

Structure of KDM1A/LSD1 (PDB ID: 6KGK) was obtained from the Protein Data Bank. Molecular docking was performed by Molecular Operating Environment (MOE) modeling software, 2019.0102. Ligand binding pocket residues were selected by graphical tools in MOE, and chemical structures of compounds were written with MOE either. In the docking calculation, potential energy maps of the receptor were calculated using default parameters. Compounds were imported into MOE. Conformational sampling was based on the Monte Carlo procedure, and finally the lowest-energy and the most favourable orientation of the ligand was selected.

### Statistical Analysis

Data were analyzed by Student’s *t* tests and log rank test for group differences, and by two-way ANOVA for condition and group differences together using GraphPad Prism software. *p < 0.05, **p < 0.01.

## Results

### Discovery of Monobenzone as a Potent and Reversible KDM1A Inhibitor

**FIGURE 1:**
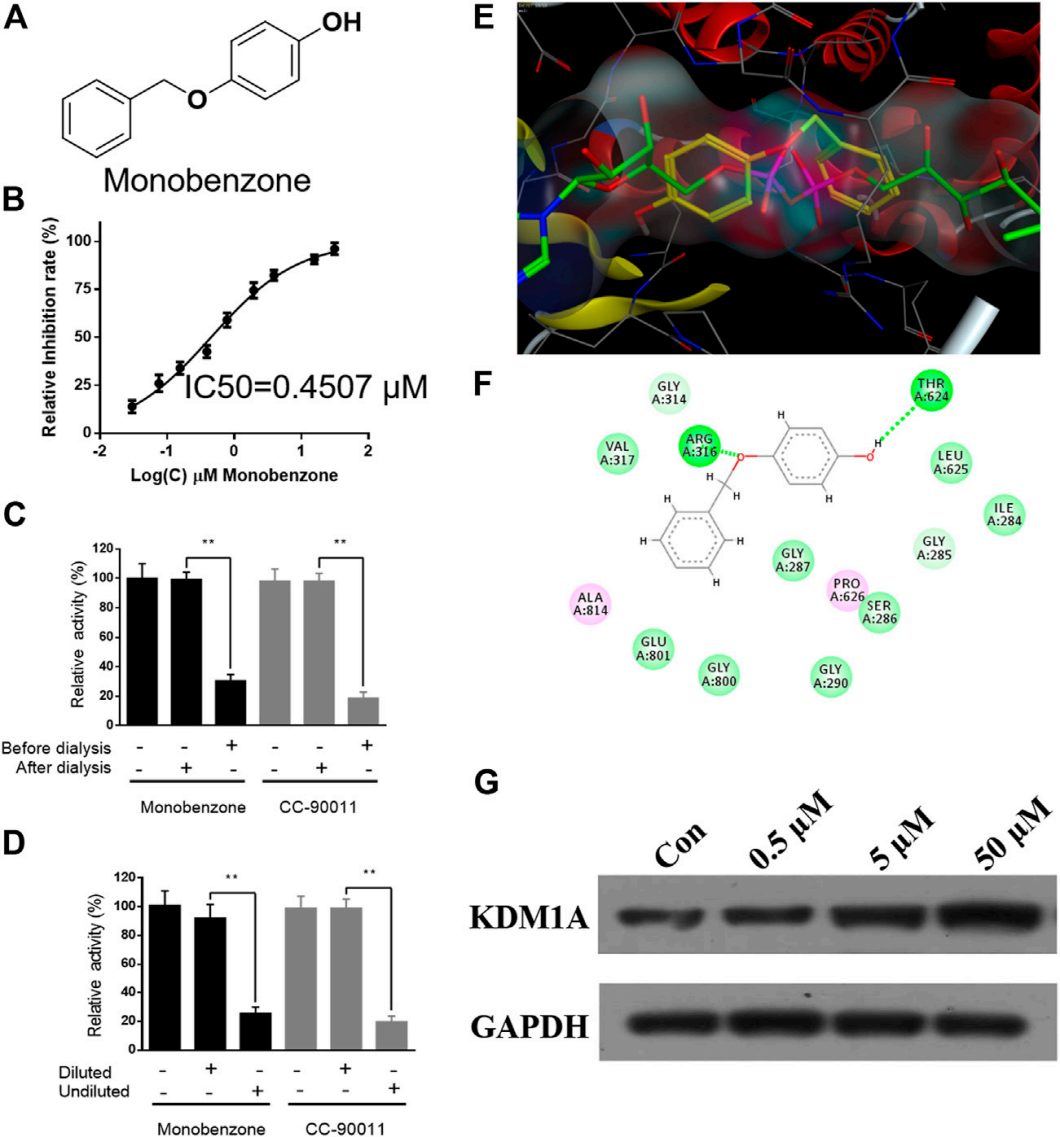
Monobenzone inhibits KDM1A in a reversible manner. **(A)** Structure of monobenzone; **(B)** Inhibition curve of monobenzone against KDM1A; **(C, D)** Dialysis **(C)** and dilution **(D)** assays of monobenzone for reversible inhibitory evaluation. Reversible inhibitor CC-90011 was used as a positive control; **(E)** Docking analysis of monobenzone in KDM1A (PDB: 6KGK), monobenzone was shown in yellow, FAD was shown in green; **(F)** Interaction between monobenzone and KDM1A was predicted by docking analysis, hydrogen bond was shown as dash line in green; **(G)** Thermal stability of KDM1A treated with monobenzone at indicated concentration and heated at 53 °C. All experiments were performed more than three times, and data were shown as mean ± standard deviation, **p < 0.01.

To discover KDM1A inhibitor with novel skeleton, an FDA approved drug library containing 1971 compounds from APExBIO was subjected to KDM1A inhibitor screening assay. In the first-round screening, all compounds were tested at 10 μM. Then, the 12 compounds with inhibitory rate more than 50% were collected and subjected to KDM1A inhibitor screening assay with H_2_O_2_ instead of KDM1A recombinant, so that false positive compound that can react with H_2_O_2_ or HRP can be excluded. After that, KDM1A inhibitory activity of all these obtained compounds were tested, and monobenzone (Figure 1A) was characterized as one of the most potent KDM1A inhibitors with IC_50_ = 0.4507 μM (Figure 1B). To evaluate the selectivity, monobenzone was subjected to monoamine oxidase A/B (MAO-A/B) assay as KDM1A is a homology of MAO-A/B, and results suggested that monobenzone failed to inhibit 5% of MAO-A/B activity even at 100 μM. To explore the binding mechanism, monobenzone was subjected to the dilution assay and dialysis assay to test the KDM1A binding reversibility, and results in Figures 1C,D indicated that when monobenzone was incubated with KDM1A recombinant at a high concentration at 30 μM, depletion of monobenzone by either 80 folds dilution or dialysis can refresh the activity of KDM1A recombinant, suggesting that monobenzone may bind and inhibit KDM1A in a reversible manner as CC-90011, which is a reversible KDM1A inhibitor in clinic trials (Dai et al., 2020). Further docking analysis using Molecular Operating Environment (MOE, 2019.0102) revealed that monobenzone may penetrate into the FAD binding cavity of KDM1A, and overlap with the position of FAD in the catalytic cavity (Figures 1E,F). Moreover, ligand interaction analysis for the docking result using Discovery Studio Visualizer 4.5 indicated that monobenzone may form two hydrogen bonds with Arg316 and Thr624, respectively. Additional cell thermal shift assay also confirmed that monobenzone can bind KDM1A in MGC-803 cells (Figure 1G). All these results gave a support that monobenzone is a potent and reversible KDM1A inhibitor.

### Monobenzone is a Cellular Active KDM1A Inhibitor

**FIGURE 2:**
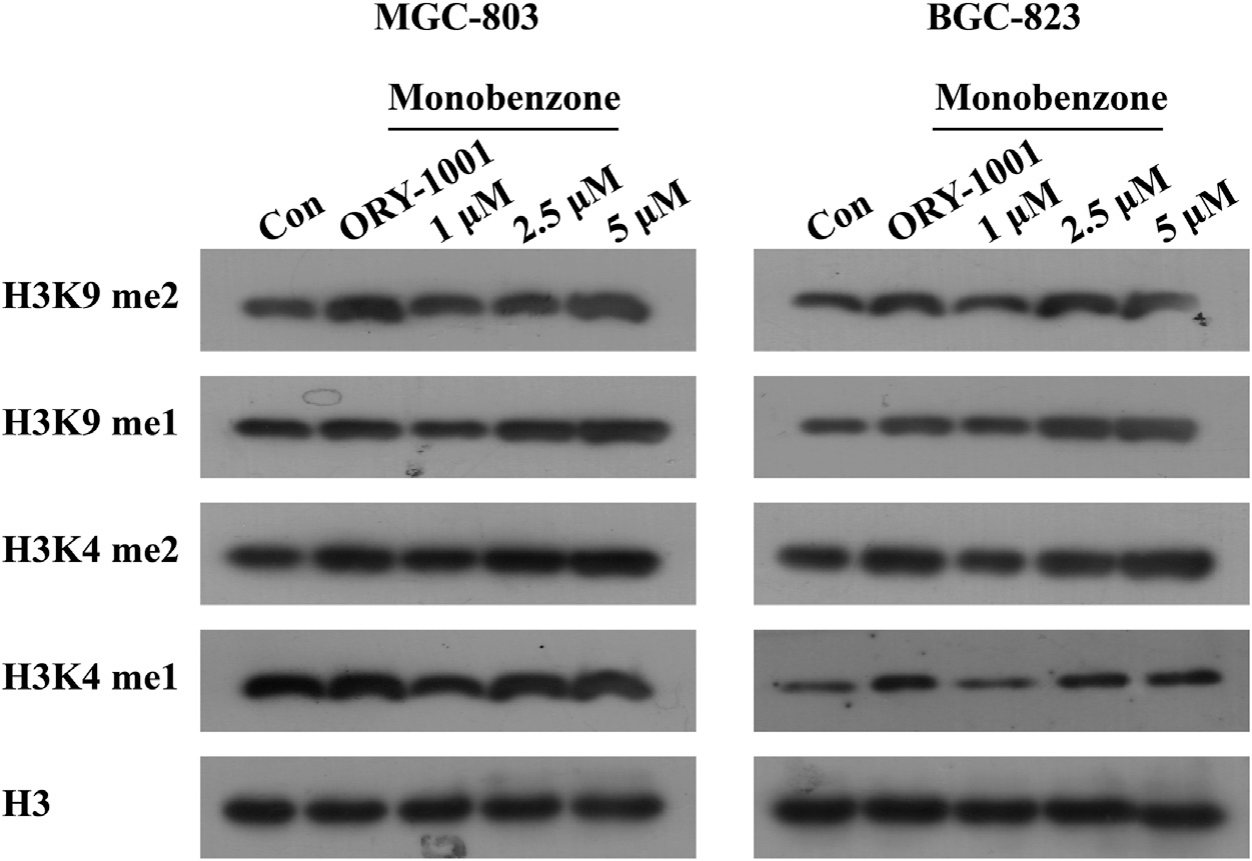
Monobenzone treatment induced the methylation levels of KDM1A substrates. The expression levels of KDM1A substrates, H3K4me1/2 and K3K9me1/2, in MGC-803 and BGC-823 cells following monobenzone treatment as indicated for 5 days, ORY-1001 was used as a positive control with 1 μM.

Once monobenzone has been characterized as a potent and reversible KDM1A inhibitor, whether monobenzone is cellular active remains unknown. To answer this question, as KDM1A was reported to contribute to gastric cancer progression (Ma et al., 2015; Dai et al., 2020; Ma et al., 2020), monobenzone was applied to gastric cancer cell lines MGC-803 and BGC-823, two LSD1 overexpressed cell lines as reported (Zheng et al., 2013; Fang et al., 2017), for 5 days treatment, respectively. Results indicated that monobenzone can inhibit MGC-803 and BGC-823 cells proliferation with IC_50_ as 7.82 ± 0.55 μM and 6.99 ± 0.51 μM, respectively. Hence, inhibitory activity of monobenzone against cellular KDM1A was further evaluated. As H3K4me1/2 and H3K9me1/2 are KDM1A substrates, and KDM1A could demethylate H3K4me1/2 and H3K9me1/2 in a FAD dependent manner (Amente et al., 2013), to evaluate whether the monobenzone can effectively inhibit the enzyme activity of KDM1A, we detected the change of KDM1A substrates after monobenzone treatment. As shown in Figure 2, monobenzone treatment for 5 days could significantly induce the accumulation of H3K4me1/2 and H3K9me1/2 in a dose dependently manner, which confirmed that the cellular activity of KDM1A was inhibited by monobenzone treatment.

### Monobenzone Inhibits Gastric Cancer Cells Migration

**FIGURE 3:**
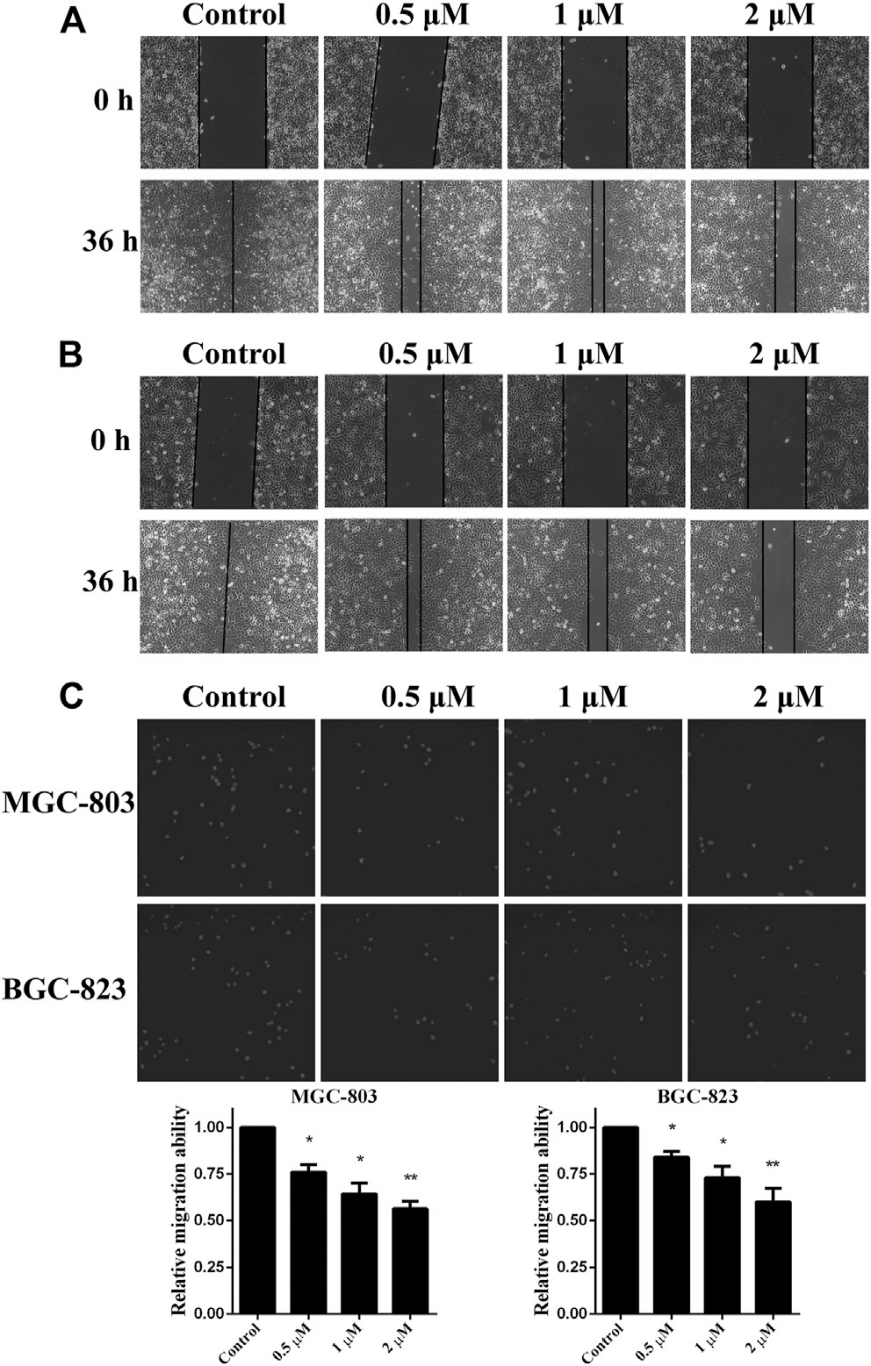
Monobenzone suppresses the migration of gastric cancer cells. **(A, B)** Images of MGC-803 **(A)** and BGC-823 **(B)** with monobenzone treatment in wound healing assay, bar = 100 μm **(C)** The migration ability of MGC-803 and BGC-823 cells following 36 h treatment of monobenzone was detected using Transwell experiment coupled with high contenting screening for cell quantification. All experiments were performed more than three times, and data were shown as mean ± standard deviation. *p < 0.05; **p < 0.01.

As *in vitro* experiments confirmed that monobenzone inhibited KDM1A enzyme activity effectively, and KDM1A plays as a contributor in the migration of gastric cancer (Pan et al., 2019; Zhang et al., 2019), we assessed the effects of monobenzone on the migration ability of gastric cancer cells. To assess the inhibitory activity of monobenzone on gastric cancer migration, wound healing assay was performed, and results in Figures 3A,B suggested that monobenzone can inhibit the wound healing in a dose dependent manner in either MGC-803 (Figure 3A) or BGC-823 (Figure 3B) cell lines. To further validate the inhibitor role of monobenzone on gastric cancer migration, transwell experiment was applied in combination with high contenting screening, and result in Figure 3C showed that monobenzone can weaken the migration ability of MGC-803 and BGC-823 cells in a concentration dependent manner either. Taken together, these experiments proved that monobenzone treatment could effectively repressed migration of gastric cancer cells.

### Monobenzone Reverses Epithelial Mesenchymal Transition in Gastric Cancer Cells

**FIGURE 4:**
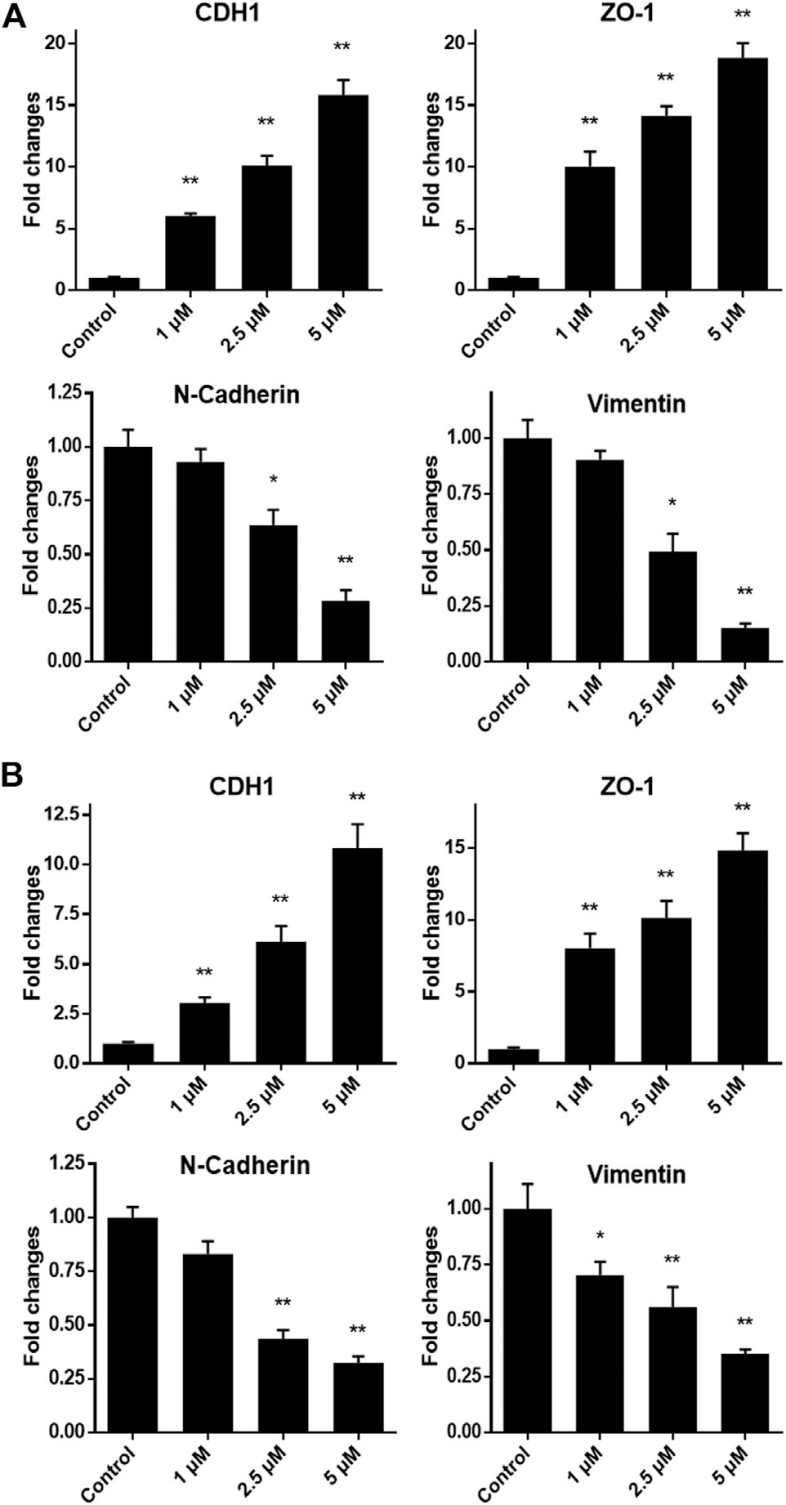
Monobenzone induces the mRNA change of EMT markers, including CDH1, ZO-1, N-Cadherin and Vimentin, in gastric cancer cell line MGC-803 **(A)** and BGC-823 **(B)**. All experiments were performed more than three times, and data were shown as mean ± standard deviation. *p < 0.05; **p < 0.01.

As we have confirmed that monobenzone treatment could effectively repress migration of gastric cancer cells, the mechanism behind is still unknown. As reported, KDM1A is a driver of epithelial–mesenchymal transition (EMT) and it can contribute to cell migration and invasion (Ambrosio et al., 2017; Boulding et al., 2018; Das et al., 2019), mRNA expressions of EMT biomarkers, including CDH1, ZO-1, N-Cadherin and Vimentin, were investigated when gastric cancer cells were exposed to different concentrations of monobenzone for 36 h. As shown in Figures 4A,B, monobenzone can significantly induce the mRNA accumulation of CDH1 and ZO-1, two epithelial cell markers; while it can also suppress the mRNA amount of N-Cadherin and Vimentin, two mesenchymal cell markers. All these data suggested that monobenzone can reverse EMT of gastric cancer cells.

## Discussions

Histone modifications play a critical role in cancer onset and progression, which have become a major focus for pharmacological cancer interventions (Audia and Campbell, 2016). Among the diverse histone modifications, histone methylation is one of the most important histone modifications. As the first identified histone demethylase, there are more and more reports have pointed out that KDM1A is a potential drug target for the treatment of cancers (Zheng et al., 2016c; Doll et al., 2018; Lee et al., 2019; Dai et al., 2020). Currently, numerous natural and synthetic KDM1A inhibitors have been identified in the last decades (Zheng et al., 2016b; Li Y. et al., 2019; Dai et al., 2020; Jia et al., 2020), and some of which are currently undergoing clinical assessment for the treatment of acute myeloid leukemia (AML), small cell lung cancer (SCLC) as well as some other solid tumors (Mohammad et al., 2015; Maes et al., 2018). However, none of them has entered into market, so, more effective and specific KDM1A inhibitors for therapeutic intervention in various cancers are still needed to be developed.

In our study, monobenzone was first characterized as a KDM1A inhibitor, it can effectively repress KDM1A activity selectively in a reversible manner in biochemical level, and additional docking analysis indicated that monobenzone may penetrate into the cavity where FAD stands in KDM1A, of which it may form two hydrogen bonds with Arg316 and Thr624 residues, respectively. Further cellular study suggested that monobenzone can induce the accumulation of H3K4me1/2 and H3K9 me1/2 in either MGC-803 or BGC-823 cells in a dose dependent manner. Specifically, 5 μM monobenzone performed similar induction effect of H3K4me1/2 and H3K9me1/2 as ORY-1001. In-depth analysis suggested that monobenzone showed strong inhibitory effect on migration ability of gastric cancer cells by reversing EMT. This finding endowed monobenzone with a new function as migration inhibitor. Based on this study, the biological application of monobenzone is no longer restricted to be a depigmentor, but indicating that monobenzone may serve as a novel skeleton for developing KDM1A inhibitors.

## Data Availability

The raw data supporting the conclusions of this article will be made available by the authors, without undue reservation.
